# Supplementary figures and images for: Finding Drug Repurposing Candidates for Neurodegenerative Diseases using Zebrafish Behavioral Profiles
Source: bioRxiv. 2023 Sep 14:2023.09.12.557235. Preprint. [Version 2] doi: 10.1101/2023.09.12.557235 (PMC10515830; doi:10.1101/2023.09.12.557235)

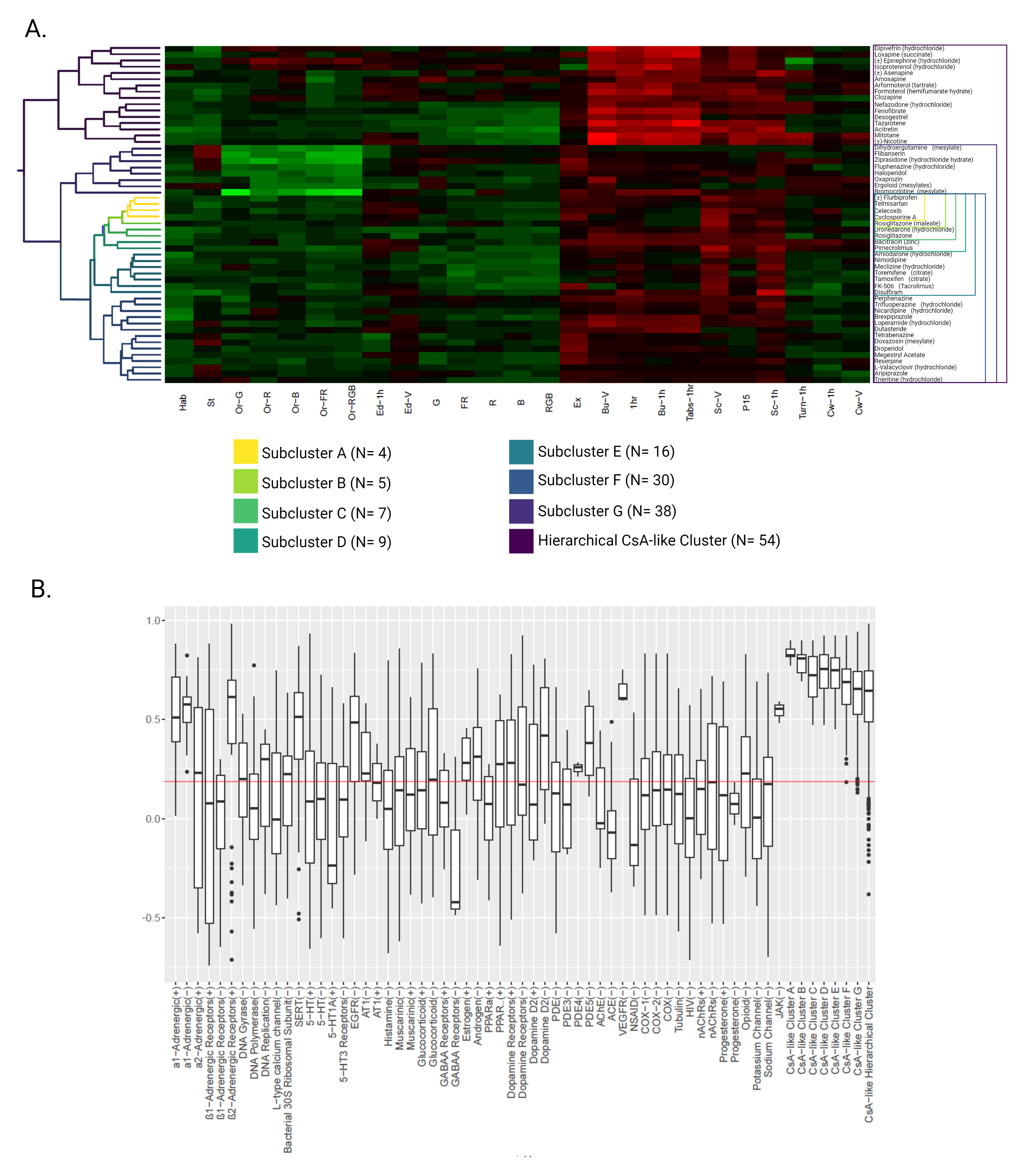

Supplement: Supplement 1 — Fig. S1. (A) Subclusters of CsA-like compounds identified with hierarchical clustering. (B) Box-whisker plots of Pearson correlation values of compounds with the same primary target; mechanisms of action are marked (+) for agonists and (−) for antagonists. Pearson correlation values for the subclusters of CsA-like compounds are also plotted. The mean correlation of biologically unrelated compounds is plotted as a red line (0.186). [file media-1.tif]

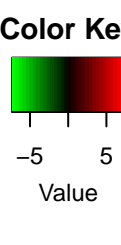

# Hierarchical Clustering

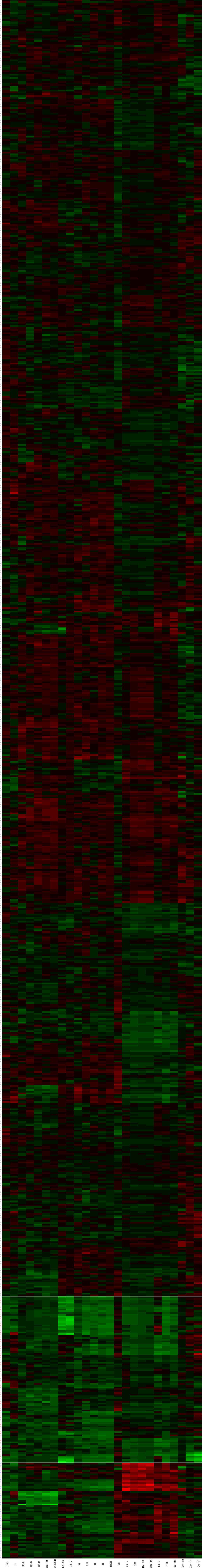

Supplement: Supplement 2 — Fig. S2. Hierarchical cluster analysis of the behavioral profiles for all screened compounds. [file media-2.pdf]

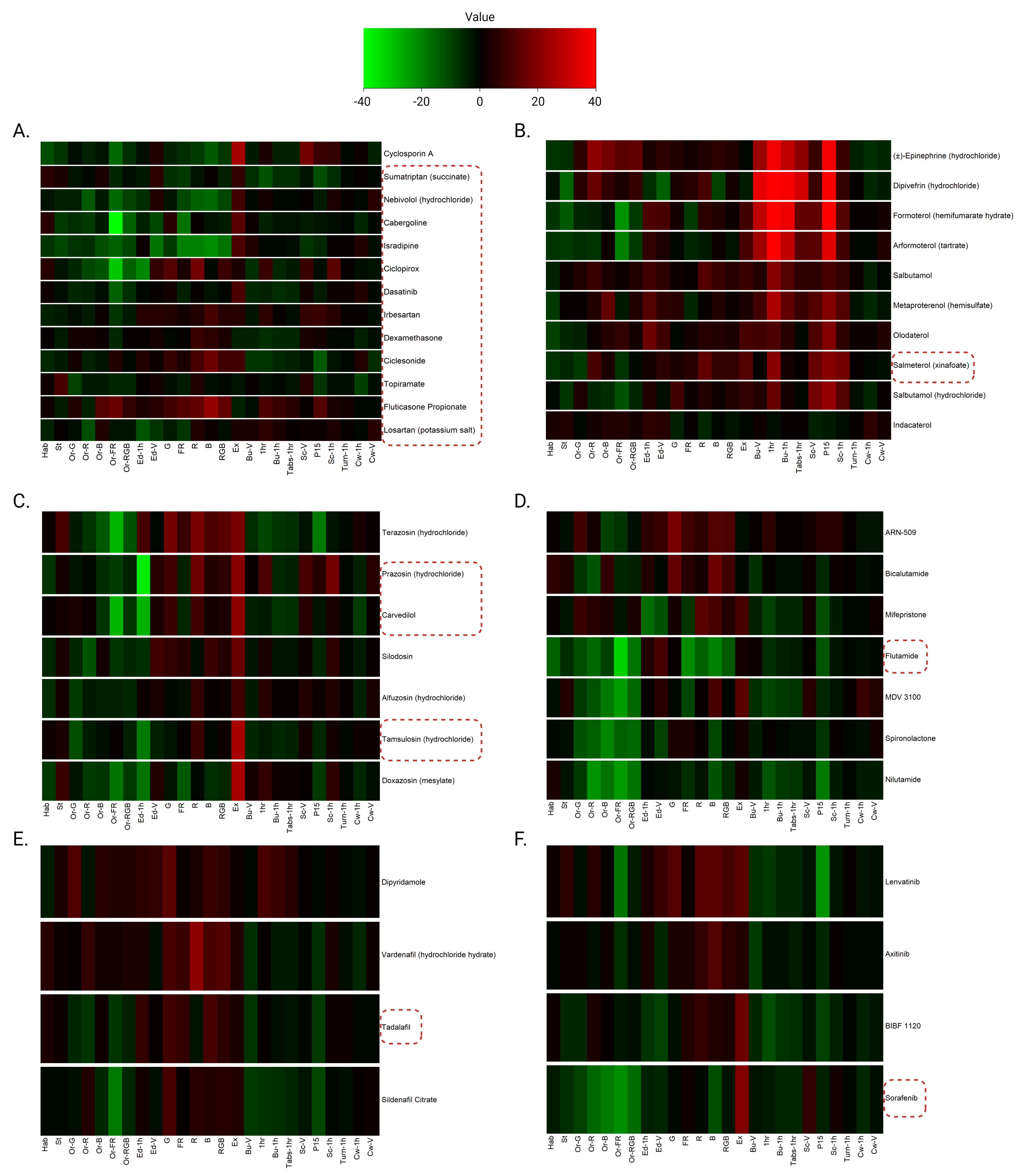

Supplement: Supplement 3 — Fig. S3. Behavioral profiles of 19 compounds previously identified as CsA-like during the screening of the Tocriscreen FDA-Approved Drugs Library. (A) 12 compounds displaying dissimilar behavioral effects from CsA. Behavioral profiles induced by biologically similar compounds, namely (B) β2-adrenergic receptor agonists, (C) α1-adrenergic receptor blockers, (D) androgen blockers, (E) PDE5 inhibitors, and (F) VEGFR inhibitors. [file media-3.tif]
